# Dose response of black American mink to Aleutian mink disease virus

**DOI:** 10.1002/iid3.290

**Published:** 2020-03-13

**Authors:** A. Hossain Farid, Irshad Hussain

**Affiliations:** ^1^ Department of Animal Science and Aquaculture, Faculty of Agriculture Dalhousie University Truro Nova Scotia Canada

**Keywords:** Aleutian mink disease virus, American mink, anti‐AMDV antibody, dose‐response relationship, viremia

## Abstract

**Introduction:**

Aleutian mink disease virus (AMDV) causes a serious health problem for mink globally. The disease has no cure nor an effective vaccine and selection for tolerance using antibody titer is adopted by many mink farmers. The objective of this study was to investigate the effects of various doses of a local AMDV isolate on the response of black American mink to infection with AMDV.

**Methods:**

Eight black American mink were each inoculated intranasally with 0.5 mL of eight serial 10‐fold dilutions (10^0^ to 10^−7^) of a 10% spleen homogenate containing a local AMDV isolate. Blood samples were collected on days 0, 20, 35, 56, 84, 140, and 196 postinoculation (dpi). Anti‐AMDV antibodies and viral DNA were tested by counter‐immunoelectrophoresis (CIEP) and PCR, respectively. Animals that were PCR or CIEP positive at 196 dpi (n = 41) were killed at 218 dpi, and samples of blood and seven organs were tested by CIEP and PCR.

**Results:**

Antibody production persisted in all seroconverted mink until the termination of the experiment, whereas 71.1% of the mink showed short‐lived viremia. Significant associations were observed between inoculum dose and the incidence of viremia until 84 dpi which disappeared thereafter, whereas associations between inoculum dose and the incidence of seropositive mink were significant on all sampling occasions. Antibody titer at 218 dpi significantly decreased with decreasing inoculum dose. AMDV DNA was detected in the bone marrow, lymph nodes, and spleen samples of almost all mink inoculated at every dose but was not detected in other organs of some mink.

**Conclusions:**

CIEP is more accurate than PCR for detecting AMDV infection in mink. Using antibody titer in naturally infected mink may not be accurate for the identification of tolerant mink.

## Graphical abstract

The predicted probabilities for viremia across all inoculum doses were the greatest at 20 dpi, and the effect became smaller as times after inoculation prolonged, and almost disappeared after 84 dpi. The predicted probabilities for viremia across all eight inoculum doses (100 to 10‐7) on the six sampling occasions (20, 35, 56, 84, 140 and 196 day post‐inoculation) were the greatest at 20 dpi, and the effect became smaller as times after inoculation prolonged, and almost disappeared after 84 dpi.

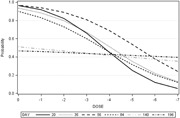

**Abbreviations**
AD: Aleutian disease
AMDV: Aleutian mink disease virus
CIEP: counter‐immunoelectrophoresis
PCR: polymerase chain reaction

## 1 INTRODUCTION

Aleutian mink disease virus (AMDV) poses a considerable health problem for mink production globally. Aleutian disease (AD) reduces reproductive performance and kit survival^1^, ^2^, ^3^ and has no cure nor an effective vaccine.^4^ Long‐term viral eradication programs have not been successful in controlling the infection in many countries.^5^, ^6^ It has long been known that some AMDV‐infected mink do not succumb to the disease and live healthy and productive lives.^7^, ^8^, ^9^, ^10^, ^11^, ^12^ It was for this reason that selection for tolerance to AD was adopted by many mink farmers in Nova Scotia, Canada, after an AMDV outbreak in 2012 and 2013 (unpublished data). Selection for tolerance to AD has also gained momentum in other counties.^3^

Defining a phenotype that can be accurately measured on live animals and has a strong association with tolerance is the first step in designing a successful selection program for establishing herds tolerant to AMDV infection. The low‐cost on‐farm iodine agglutination test was successfully used in establishing tolerant mink herds in Nova Scotia.^13^ Recently, enzyme‐linked immunosorbent assays were developed for measuring antibody titer in mink infected with AMDV^14^, ^15^ and have been used to identify animals with low antibody titers,^3^ which are expected to be able to tolerate AD. The characteristics of animal response to infection need to be clearly understood before enzyme‐linked immunosorbent assays or iodine agglutination test can effectively be used in assessing the degree of tolerance of mink to AMDV infection.

Mink are commonly evaluated for tolerance on naturally infected farms where the most likely scenario is exposure to low doses of the virus as a result of the slow rate of horizontal AMDV transmission.^11^, ^12^, ^16^ Although all mink on chronically infected farms ultimately become exposed to the virus, the time for the establishment of infection under natural conditions is unpredictable. Furthermore, to establish infection, the amount of virus intake must overwhelm the host's defense mechanism. The immune system of some mink seems to be capable of curtailing the establishment of infection or producing detectable levels of antibodies when exposed to low viral doses,^17^, ^18^, ^19^ which is manifested as seronegative mink on chronically infected farms.^9^, ^12^, ^13^, ^20^ It is important to know whether the presence of seronegative mink on infected farms was the result of genetic differences or because the level of the virus to which they were exposed was too low. Interestingly, when pairs of littermates were kept in the same cage for several months, only one turned seropositive in some cages,^9^, ^12^ which could suggest that viral dose might have been a more determining factor than animal genetic makeup. It is thus important to understand the effects of various doses of any AMDV strain on antibody titer and viremia at the time when replacement mink are selected for tolerance (November and December).

The rationale behind the present study was to investigate the effects of various doses of a moderately pathogenic local AMDV isolate^21^ on the response of black American mink in Nova Scotia which carry the dominant Jet‐black allele.^22^ The second objective was to estimate the dose of the inoculum that provokes infection in 50% of mink (ID_50_). This estimate is needed before planning an experiment to assess the animals' degree of tolerance to this virus. Intranasal inoculation following sedation was used in this study because it was found to be an efficient method of establishing infection in mink without destroying the animals' physical barriers,^21^ and it approximates natural exposure to the virus.

## 2 MATERIALS AND METHODS

### 2.1 Statement of animal care

All protocols were performed according to the standards of the Canadian Council for Animal Care (http://www.ccac.ca) after approval by the institutional Animal Care and Use Committee (File #2009‐014). Animals were sedated before inoculation, blood sampling, and euthanasia, as previously explained.^23^ Euthanasia was performed in sedated mink by intracardial injection of sodium pentobarbital (Euthanyl, Bimedia‐MTC, Cambridge, ON, Canada) at the dose of 100 mg per kg body weight.

### 2.2 Source of the virus

The viral inoculum was a 10% (w/v) passage 2 of a local strain of AMDV prepared from the spleens of 57 mink harvested 10 days postinoculation (dpi) and stored at −80°C, as previously described.^23^ The inoculum was thawed at room temperature and serially diluted with sterile phosphate‐buffered saline on the day of inoculation.

### 2.3 Source of animals and experimental procedure

A total of 34 juvenile (3‐month old) and 34 adult (15‐month old) female black American mink (*Neovison vison*) from Dalhousie University Fur Unit, which has been AMDV‐free for many years, were transferred to a biosecure building (Aleutian Disease Research Center). Housing and management of the animals were previously reported.^23^ Animals were divided within age to eight groups of eight each, were sedated and 0.25 mL of each of the eight 10‐fold serially diluted spleen homogenate (from 10^0^ to 10^−7^) was deposited into each of their nostrils using a 1‐mL syringe without a needle. Inoculation was performed after 1 week of adaptation to the new environment, during which the diet gradually changed from a wet to a commercial dry feed (National Feeds Inc., Maria Stern, OH, USA). Four mink were kept as uninoculated controls, which were housed three cages apart from inoculated animals, fed first daily, and were sampled first to minimize the chance of cross‐contamination.

### 2.4 Sampling

Blood samples were collected by toenail clipping after sedation at 0 (before exposure), 20, 35, 56, 84, 140, and 196 dpi in heparinized capillary tubes for the CIEP test and in ethylenediaminetetraacetic acid‐coated capillary tubes for viral detection by polymerase chain reaction (PCR). The saliva and rectal swabs were collected on the six sampling occasions from 35 mink which received greater than 10^−4^ doses, and samples were processed as detailed in a previous report.^23^ The mink that were inoculated with 10^−4^ or higher doses (n = 36) and those which were inoculated with lower than 10^−4^ doses but were PCR or CIEP positive at any sampling occasions (n = 5) were sedated at 218 dpi and blood samples were collected by cardiac puncture for CIEP and PCR tests. These animals were then euthanized, pelted, and samples of the spleen, bone marrow, lungs, liver, kidneys, heart, mesenteric lymph nodes, and small intestine (duodenum) were collected aseptically. Tissue samples, except blood, were kept frozen at −80°C until use. The pelts were closely evaluated for the presence of white hair fibers (sprinklers) on 196 and 218 dpi and scored from zero (no white hair) to 4 (large number of white hair fibers).

### 2.5 Laboratory procedures

The blood and tissue samples were processed for the CIEP and PCR tests, as previously explained.^23^ The CIEP test^3^ was performed on plasma and cell‐free suspensions of six organs (spleen, lymph nodes, liver, kidneys, lungs, and intestine) by the Animal Health Laboratory of the Nova Scotia Department of Agriculture in Truro, Nova Scotia, Canada, which is accredited for this test by the Standards Council of Canada. Bone marrow samples were not tested by CIEP because it was flushed out of the tibia with 0.5 mL of phosphate‐buffered saline, causing inaccuracies in its antibody titer. The cell‐cultured antigen was obtained from the Research Foundation of the Danish Fur Breeders Association. In addition to the fresh plasma samples that were tested by CIEP, frozen plasma samples collected on 218 dpi were thawed, twofold serially diluted 10 times (1/2 to 1/1024) and tested by CIEP in duplicate. The titer of anti‐AMDV antibodies was recorded as the reciprocal of the highest dilution of plasma, which generated a positive or a faint‐band. Infectious materials were handled in a biosafety level 2 laboratory following the approved Standard Operating Procedures.

DNA was extracted from 200 μL of cell‐free suspensions of the seven organs, saliva, and rectal preparations and from 50 to 120 μL of plasma using Dynabeads Silane viral nucleic acid extraction kit (Invitrogen, Burlington, ON, Canada) and eluted in 100 μL of elution buffer. DNA was amplified by PCR using primers 60F and 60R, as previously described.^24^ Three PCR tests were performed on each sample using 1.5, 2.5, and 3.5 μL of DNA solution in 15 μL total volumes. In cases where the results were negative or inconclusive, the tests were repeated. Sample preparation, PCR cocktail preparation, PCR amplification, and PCR product testing were performed in four separate laboratories with unidirectional sample movement to avoid cross‐contamination. Sterile filtered‐tips were used throughout the experiment. Histologic lesions on liver and kidneys of dead animals were subjectively scored by an experienced pathologist on a scale of 0 (no lesion) to 4 (very severe lesions from advanced AD). Scoring was based on the accumulation of plasma cells in the tissues with associated lesions.^25^

### 2.6 Data analysis

Data were analyzed using SAS, version 9.4 for Windows (SAS Institute, Cary, NC). There were few inconclusive PCR results that were considered as positives. Binary logistic regression models were used to estimate the effects of inoculum dose and age of mink on the incidence of CIEP and PCR‐positive blood and organs within each sampling occasion. Log of odds (β), odds ratio (e^β^), and the predicted probability of seroconversion and viremia (y = *e* ^intercept + β*dose^/(1 + *e* ^intercept + β*dose^)) was calculated across inoculum doses for each sampling occasion. Before analyses, antibody titers were log‐transformed as log_2_(titer) = 0 if titer = 0 and log_2_(titer) + 1 if titer > 0.

The proportion of positive PCR and CIEP organs was compared using a generalized estimating equation (GEE) algorithm and the independent correlation structure in the GENMOD procedure with a binomial distribution and the logit link function. The model included the fixed effects of age and organ. The random effect of individual mink was used in the REPEATED statement to take care of the correlation in the prevalence of the PCR and CIEP results among organs of the same mink. Least square means and their standard errors were reported after conversion to the original scales by the iLink option, and multiple comparison of means was performed using Tukey's adjustment. The effect of the age of mink was not significant and was deleted from the final analysis. The number of animals that were inoculated with 10^−5^, 10^−6^, and 10^−7^ doses and were killed at 218 dpi was small and data of these animals were combined before analyses.

Sensitivity and specificity of plasma CIEP relative to the corresponding PCR results and those of PCR results of saliva and rectal swabs relative to plasma PCR were calculated using the Vassarstats software (http://vassarstats.net/clin1.html). Agreements between CIEP and PCR results of plasma in the same animals and between PCR results of plasma, saliva, and rectal swabs were calculated by the kappa coefficient. The *P* values in this analysis do not measure the strength of agreements, rather they test whether the estimated kappa coefficient is not due to chance. Associations among the incidence of sprinklers and antibody titer were calculated by Spearman's rank correlation. The Probit procedure with the logistic distribution was used to calculate 50% infectious dose (ID_50_) of the 10% spleen homogenate using CIEP and PCR results at 20, 35, and 56 dpi.

## 3 RESULTS

### 3.1 Viral DNA in plasma

Six inoculated mink died during the course of the study, one from each of the 10^0^, 10^−4^, 10^−6^, and 10^−7^ doses and two from 10^−3^ dose (Table 1). Four of the dead mink were PCR and CIEP positive from 20 or 35 dpi until death and showed minor (score 1) to severe (score 3) AD lesions on their kidneys and/or livers (Table 1). One mink in each of the 10^−4^ and 10^−6^ doses remained CIEP and PCR negative until death and did not show any AD lesions at necropsy.

**Table 1:** Distribution of dead mink by inoculum dose, PCR, and CIEP results and sampling days, and AD lesion scores at necropsy

Dose	Days postinoculation^a^					AD lesion scores^b^	
	20	35	56	84	140	Kidneys	Liver
0	+/+	+/+	+/+	.	.	1	2
−3	+/+	+/+	.	.	.	0	1
−3	+/–	+/+	+/+	+/+	.	2	3
−4	–/–	–/–	–/–	–/–	.	0	0
−6	–/–	–/–	–/–	–/–	–/–	0	0
−7	+/–	+/+	+/+	+/+	.	2	1
Control	–/–	–/–	–/–	–/–	.	0	0

Abbreviations: AD, Aleutian disease; CIEP, counter‐immunoelectrophoresis; PCR, polymerase chain reaction.

^a^ PCR result/CIEP result, “+” positive, “–“ negative, “.” animal was dead.

^b^ 0, 1, 2, 3 are none, minor, moderate, and severe lesions, respectively.

**Figure 1:**
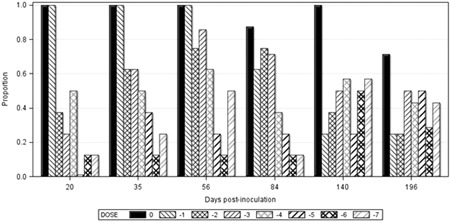
Proportion of PCR‐positive plasma by inoculum dose on different days postinoculation

**Figure 2:**
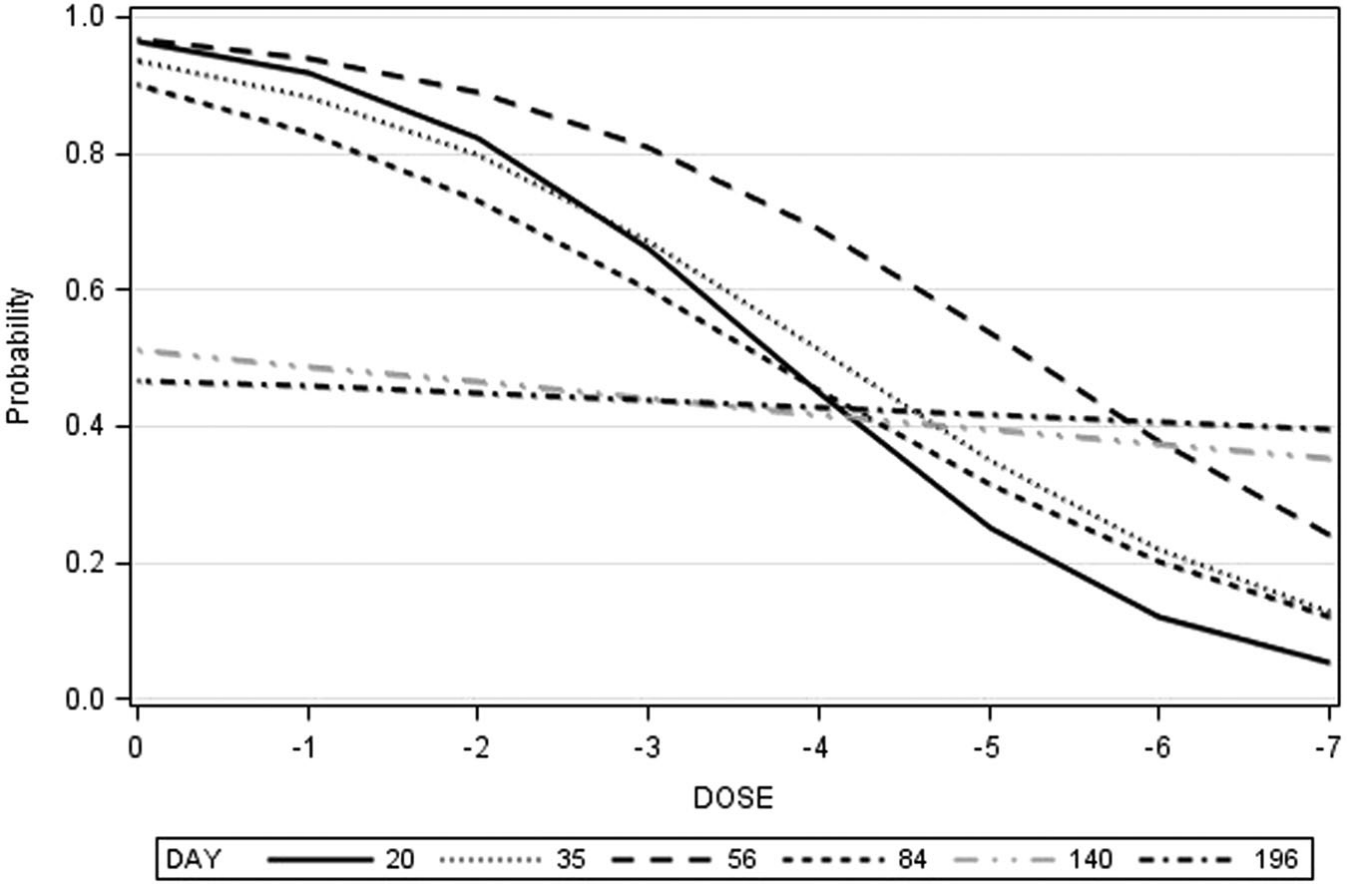
Probability of PCR‐positive plasma by inoculum dose on different days postinoculation

All mink inoculated with the 10^0^ or 10^−1^ doses were PCR positive by 20 dpi, whereas some mink inoculated with the other doses remained PCR negative until 196 dpi (Figure 1). Seven mink became PCR positive for the first time at 196 dpi and six mink (four juveniles and two adults) remained PCR negative until 196 dpi; one in each of the 10^−2^, 10^−5^, 10^−6^, 10^−7^, and two in the 10^−4^ doses. Four of the six PCR negative mink at 196 dpi, which were inoculated with 10^−5^ or higher doses and were kept until 218 dpi, became PCR positive at that time. Eight mink (five juveniles and three adults) and three juveniles, which became viremic at 20 or 35 dpi, respectively, remained viremic until 196 dpi, whereas viremia in 32 of the 45 mink (71.1%) that became PCR positive before and survived until 196 dpi, was irregular or short‐lived. Logistic regression analyses revealed that the effects of viral dose on the incidence of viremia were significant until 84 dpi, but not at the subsequent sampling occasions. For each unit change in dose (10 times dilution), the expected change in log of odds for viremia at 20 dpi, adjusted for the age of mink, was 0.88, and gradually decreased at subsequent sampling occasions (Table 2). The estimate of odds ratio showed that the odds of viremia changed by 2.41 times for each unit change of inoculum dose at 20 dpi, and the estimates steadily decreased to 1.043 at 196 dpi. The predicted probabilities for viremia across all inoculum doses were the greatest at 20 dpi and the effect became smaller as times after inoculation prolonged, and almost disappeared after 84 dpi (Figure 2). The effects of age on the incidence of viremia were significant only at 20 dpi. The log of odds for viremia was lower in the juvenile than in the adult mink at 20 dpi (Wald *χ* ^2^_(1)_ = 4.78, *P* = .03). This odds ratio suggests that the odds for juvenile mink becoming viremic, adjusted for inoculum dose, was 0.19 as high as that of the adult mink (Table 2).

**Table 2:** Logistic regression of the incidence of PCR‐positive plasma on inoculum dose and age of mink for each sampling occasions

Days postinoculation	Intercept ± SE	Dose		Mink age	
		*β* ± SE^a^	Odds ratio	*β* ± SE^a^	Odds ratio
20	3.311 ± 0.924	0.880 ± 0.212**	2.410	−1.659 ± 0.758*	0.190
35	2.698 ± 0.772	0.662 ± 0.165**	1.938	0.000 ± 0.000	1.000
56	3.385 ± 0.901	0.647 ± 0.170**	1.910	−0.554 ± 0.641	0.575
84	2.190 ± 0.716	0.595 ± 0.157**	1.813	−0.681 ± 0.619	0.506
140	0.048 ± 0.535	0.094 ± 0.116	1.652	0.502 ± 0.529	1.652
196	−0.126 ± 0.540	0.042 ± 0.117	1.043	−0.162 ± 0.535	0.851

Abbreviation: PCR, polymerase chain reaction

^a^ Log(odds) ± standard error.

* Significantly different from zero at *P* < .05.

** Significantly different from zero at *P* < .01.

### 3.2 Antibody in plasma

**Figure 3:**
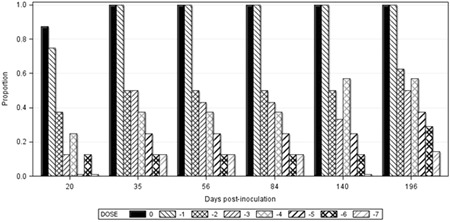
Proportion of seropositive mink by inoculum dose on different days postinoculation

**Figure 4:**
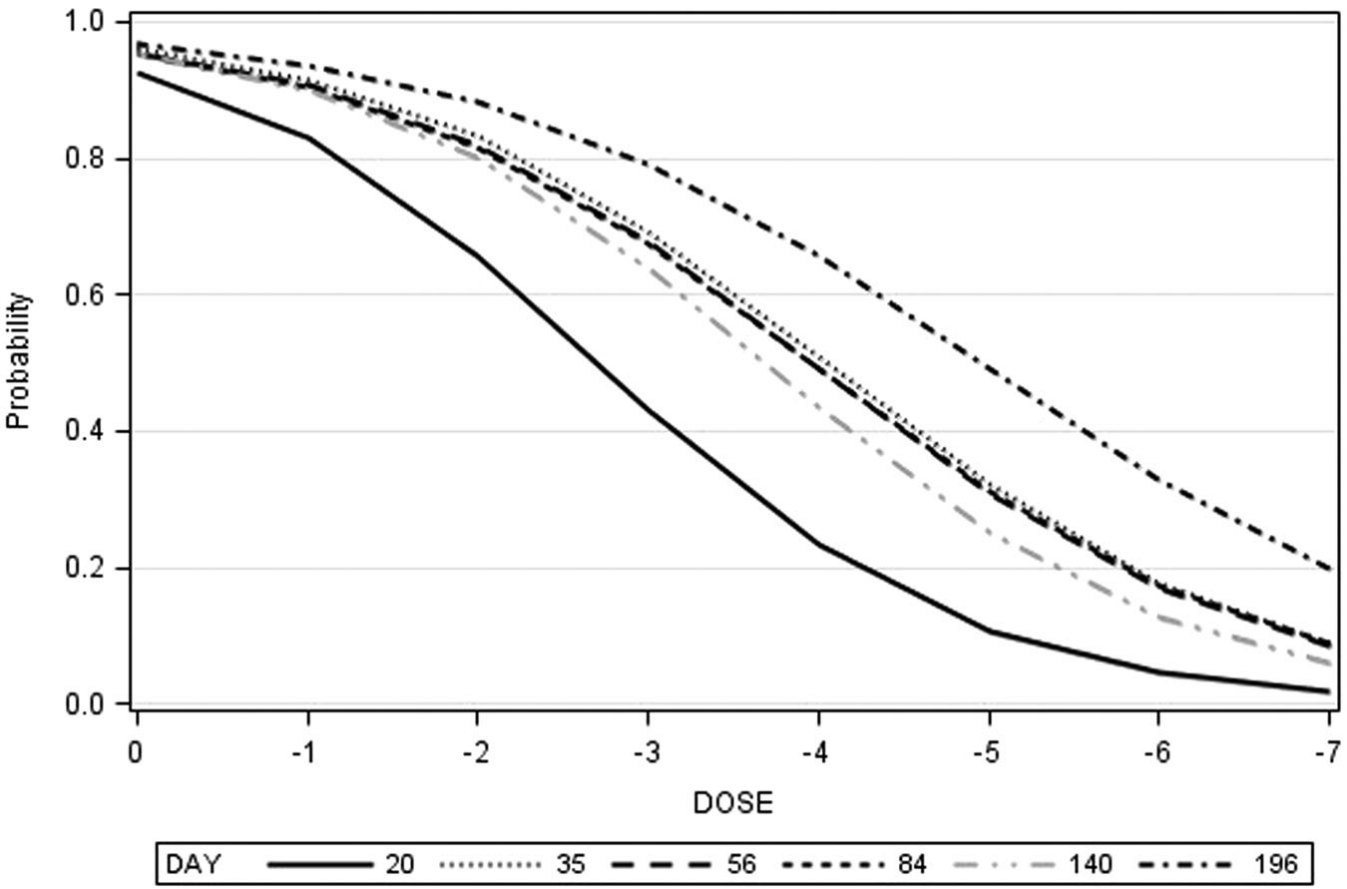
Probability of seropositive mink by inoculum dose on different days postinoculation

At 20 dpi, the incidence of CIEP‐positive cases was the highest in animals inoculated with the 10^0^ dose (0.875), and none of the mink inoculated with 10^−5^ or 10^−7^ doses were seropositive at this sampling occasion (Figure 3). All mink inoculated with the 10^0^ and 10^−1^ doses seroconverted by 35 dpi, whereas 43.1% of the 58 mink that survived until 196 dpi remained seronegative, which included three mink in each of the 10^−2^, 10^−3^, and 10^−4^ doses, five in each of the 10^−5^ and 10^−6^, and six in the10^−7^ dose. Three of the nine seronegative mink inoculated with 10^−4^ and higher doses, which tested at 218 dpi, became seropositive for the first time. Detectable levels of antibodies persisted in plasma of all seroconverted mink until the termination of the experiment. Logistic regression analyses showed that the effects of viral dose on the incidence of seroconversion were significant at all sampling occasions. For each unit change in dose, the expected change in log of odds for seroconversion at 20 dpi was 0.928, which gradually decreased on subsequent sampling occasions (Table 3). The estimate of odds ratio suggests that the odds of seroconversion changed by 2.529 times for each unit change of inoculum dose at 20 dpi and the estimates decreased by time. The predicted probabilities for seroconversion across inoculum doses were the highest at 20 dpi, but the effect of inoculum dose decreased as times after inoculation prolonged (Figure 4). The effect of age on the incidence of CIEP‐positive plasma samples was significant only at 20 dpi (Wald *χ* ^2^_(1)_ = 3.98, *P* = .04). The odds of juvenile mink being seropositive was 0.209 times the odds of adults being seropositive at 20 dpi, but differences between the two age groups were smaller and nonsignificant at the subsequent sampling occasions (Table 3).

**Table 3:** Logistic regression of the incidence of CIEP‐positive plasma on inoculum dose and age of mink for each sampling occasion

Days postinoculation	Intercept ± SE	Dose		Mink age	
		*β* ± SE^a^	Odds ratio	*β* ± SE^a^	Odds ratio
20	2.515 ± 0.843	0.928 ± 0.238**	2.529	−1.565 ± 0.785*	0.209
35	3.171 ± 0.864	0.784 ± 0.187**	2.190	−1.071 ± 0.683	0.343
56	3.065 ± 0.859	0.774 ± 0.185**	2.169	−0.993 ± 0.685	0.370
84	3.019 ± 0.860	0.762 ± 0.186**	2.144	−1.007 ± 0.683	0.365
140	3.050 ± 0.876	0.827 ± 0.200**	2.287	−0.674 ± 0.711	0.510
196	3.374 ± 0.918	0.682 ± 0.181**	1.978	−1.195 ± 0.695	0.303

Abbreviation: CIEP, counter‐immunoelectrophoresis.

^a^ Log (odds) ± standard error.

* Significantly different from zero at *P* < .05.

** Significantly different from zero at *P* < .01.

The estimates of the sensitivity of CIEP relative to PCR in plasma varied among different sampling occasions and ranged between 0.62 (140 dpi) and 0.86 (84 dpi). The estimates of relative specificity of CIEP tests were 100% at 20 dpi but steadily decreased to 0.59 at 196 dpi (Table 4). The percentages of mink that had both PCR‐ and CIEP‐positive plasma were low at 20 dpi (31.2%), reached a peak at 35 dpi (46.9%), and then declined. The percentages of mink with both negative PCR and CIEP were the highest at 20 dpi (57.8%), then declined over time with no clear pattern. The number of cases where the two measurements agreed (both positive or negative) was over 80% until 84 dpi and dropped to 64.3% and 67.3% at 140 and 196 dpi, respectively, as reflected in the estimates of kappa coefficients.

**Table 4:** Sensitivity and specificity of plasma CIEP and of PCR results for saliva and rectal swabs relative to plasma PCR on different sampling occasions

	Number	Sensitivity^a^	Specificity^b^	+/+^c^	–/–^d^	Kappa
Plasma CIEP						
20	64	0.74	1.0	31.2	57.8	0.77
35	64	0.83	0.96	46.9	42.2	0.78
56	63	0.73	0.96	46.0	34.9	0.62
84	62	0.86	0.85	38.7	46.8	0.71
140	59	0.62	0.67	30.5	33.9	0.29
196	58	0.79	0.59	32.8	34.5	0.36
Saliva PCR						
20	35	0.85	0.00	65.7	0.0	−0.18
35	35	0.59	1.00	57.1	2.9	0.08
56	34	0.79	0.50	76.5	2.9	0.14
84	32	0.63	0.75	46.9	18.8	0.29
140	30	0.17	0.75	10.0	30.0	−0.07
196	31	0.20	0.94	9.7	48.4	0.14
Rectal PCR						
20	35	0.52	1.0	40.0	22.9	0.33
35	35	0.71	1.0	68.6	2.9	0.13
56	34	0.81	1.0	76.5	5.9	0.34
84	33	0.84	0.75	63.6	18.2	0.54
140	31	0.67	0.85	38.7	35.5	0.49
196	31	0.53	0.94	25.8	48.4	0.19

Abbreviations: CIEP, counter‐immunoelectrophoresis; PCR, polymerase chain reaction.

^a^ Sensitivity = (true positive)/(true positive + false negative).

^b^ Specificity = (true negative)/(true negative + false positive).

^c^ CIEP positive and PCR positive.

^d^ CIEP negative and PCR negative.

### 3.3 Antibody titer

Antibody titer in animals that were inoculated with10^0^ and 10^−1^ doses ranged between 128 and 1024 and those inoculated with lower doses showed a wider range (0 to 1024). Regression of log_2_ antibody titer on inoculation dose was linear and significant, but the model explained a rather small proportion of variations in log_2_ antibody titer (19.4%). Each 10‐fold increase in inoculum dose resulted in 0.992 increase in log_2_ antibody titer (Table 5). The predicted log_2_ antibody titers were 9.10 at the highest dose of the inoculum (10^0^) and 2.16 at the lowest dose (10^−7^). Animals that became PCR positive at later times after inoculation tended (*P* = .08) to have lower log_2_ antibody titer at 218 dpi (*β* = −.0084/day), but the effect of the day that animals became CIEP positive on antibody titer was negligible (Table 5). Another evidence for the effect of duration of infection on antibody titer at 218 dpi is the lower percentage of PCR‐positive plasma samples in mink with antibody titers lower than 16, compared with those with antibody titer higher than 64 (Table 6). Differences were significant on all sampling occasions except at 196 dpi. The effect of age of mink on the above‐mentioned analyses was negligible and was excluded from the final models.

**Table 5:** Regression of log_2_ (antibody titer) on inoculum dose and days postinoculation for animals that showed CIEP or PCR‐positive plasma^a^

Independent variable	Number	Intercept ± SE	*β* ± SE	Pr.	*R* ^2^, %
Dose	41	9.103 ± 0.910	0.9920 ± 0.3044	0.00	19.4
Day postinoculation					
When CIEP was positive	30	9.026 ± 0.308	−0.0023 ± 0.0049	0.64	0.8
When PCR was positive	30	9.269 ± 0.287	−0.0084 ± 0.0045	0.08	10.7

Abbreviations: CIEP, counter‐immunoelectrophoresis; PCR, polymerase chain reaction.

^a^ Excluding animals which remained PCR or CIEP negative.

**Table 6:** Percentage of PCR‐positive plasma samples on various sampling occasions by the range of antibody titer measured at 218 days postinoculation

Titer range^a^	Days postinoculation					
	20	35	56	84	140	196
0‐16	8.3	25.0	58.3	25.0	25.0	33.3
64‐1024	75.9	89.7	89.7	79.3	62.1	51.7
*χ* ^2^_(1)_	17.3	16.8	4.9	10.8	4.8	1.2
Probability	0.00	0.00	0.03	0.00	0.03	0.28

^a^ There were 12 and 29 mink in 0‐16 and 64‐1024 groups, respectively. No animal had antibody titer of 32.

### 3.4 Viral DNA and antibody in organs

AMDV DNA was detected at 218 dpi in the bone marrow, lymph node, and spleen samples of all mink inoculated with every inoculum dose, except in five spleen samples of mink inoculated with lower than 10^−1^ doses. Viral DNA was not detected in the liver, kidneys, lungs, and intestine samples of some mink inoculated with each of the doses, except in the kidneys and lungs of mink inoculated with 10^−2^ and 10^−3^ doses, respectively, where all animals were PCR positive (Table 7). Logistic regression analysis showed that the increase in inoculum dose resulted in increases in the odds of becoming PCR positive for all organs at 218 dpi, but the effect was significant only for the liver (Wald *χ* ^2^ = 4.12, *P* = .04). For each unit change in dose, the expected change in log odds for PCR‐positive liver samples was 0.407, and the estimated odds ratio suggests that the odds of PCR‐positive liver samples changed by 1.503 times for each unit change of the inoculum dose (Table 8). The predicted probabilities for PCR‐positive liver samples are 0.73, 0.65, 0.55, 0.45, 0.35, 0.26, 0.19, and 0.14 for 10^0^ to 10^−7^ doses, respectively.

**Table 7:** Incidence of PCR‐ and CIEP‐positive organs at 218 dpi by inoculum dose

Organ	10^0^	10^−1^	10^−2^	10^−3^	10^−4^	10^<−4^ ^a^
Viral DNA (PCR)						
Spleen	1.00	1.00	0.75	1.00	0.71	0.80
Lymph nodes	1.00	1.00	1.00	1.00	1.00	1.00
Bone marrow	1.00	1.00	1.00	1.00	1.00	1.00
Liver	0.86	0.50	0.50	0.50	0.43	0.20
Kidneys	0.71	0.88	1.00	0.67	0.86	0.60
Lungs	0.71	0.88	0.88	1.00	0.57	0.60
Intestine	0.86	0.50	0.63	0.67	0.57	0.20
Antibody (CIEP)						
Spleen^b^	1.00	1.00	0.63	0.50	0.57	0.40
Lymph nodes	1.00	1.00	0.57	0.50	0.57	0.40
Intestine	1.00	0.87	0.63	0.50	0.57	0.40
Number of samples^c^	7	8	8	6	7	5

Abbreviations: CIEP, counter‐immunoelectrophoresis; PCR, polymerase chain reaction.

^a^ Animals inoculated with 10^−4^ and lower doses were combined.

^b^ CIEP results were the same in the spleen, liver, kidneys, and lungs of all mink.

^c^ 1, 2, and 1 lymph node samples in 10^0^,10^−1^, and 10^−2^ doses, respectively, had missing values.

**Table 8:** Logistic regression of incidence of PCR‐ and CIEP‐positive organs on inoculum dose

Organ	Intercept ± SE^a^	*β* ± SE^a^	Wald *χ* ^2^ (Probability)	Odds ratio
PCR^b^				
Spleen	2.9373 ± 0.9800	0.3430 ± 0.2646	1.68 (0.19)	1.409
Liver	1.0158 ± 0.5745	0.4072 ± 0.2007*	4.12 (0.04)	1.503
Kidney	2.0164 ± 0.7335	0.2295 ± 0.2177	1.11 (0.29)	1.258
Lung	2.0136 ± 0.7211	0.2827 ± 0.2126	1.77 (0.18)	1.327
Intestine	1.1824 ± 0.5848	0.3407 ± 0.1927	3.13 (0.08)	1.406
CIEP				
Spleen^c^	2.6289 ± 0.8253	0.6404 ± 0.2452**	6.82 (0.01)	1.897
Lymph nodes	2.3712 ± 0.8349	0.5824 ± 0.2448*	5.66 (0.02)	1.790
Intestine	2.1846 ± 0.7331	0.5363 ± 0.2240*	5.73 (0.02)	1.710

Abbreviations: CIEP, counter‐immunoelectrophoresis; PCR, polymerase chain reaction.

^a^ Log(odds) ± standard error.

^b^ Lymph nodes and bone marrow were PCR positive in all inoculum doses and were excluded from the analysis.

^c^ CIEP results were the same in the spleen, liver, kidneys, and lungs of all mink.

* Significantly different from zero at *P* < .05.

** Significantly different from zero at *P* < .01.

Anti‐AMDV antibodies were detected in all six organs (spleen, lymph nodes, liver, kidneys, lungs, and intestine) of all mink inoculated with the 10^0^ and 10^−1^ doses, except for the intestine sample of one mink inoculated with 10^−1^ dose (Table 7). The incidence of CIEP‐positive organs of mink inoculated with the lower than 10^−1^ doses ranged between 0.63 to 0.40, and CIEP‐positive organs were almost always belonged to the same mink. Logistic regression showed that increases in inoculum dose resulted in significant increases in the odds of becoming CIEP positive in all organs. For each unit change in dose, the expected change in log odds for CIEP‐positive spleen, liver, kidney, and lung samples was 0.64, and the estimate of odds ratio suggests that the odds of CIEP‐positive samples changed by 1.897 times for each unit change of the inoculum dose (Table 8). The effect of inoculum dose on the incidence of CIEP‐positive lymph nodes and intestine samples were somewhat lower, with odds ratios of 1.79 and 1.71, respectively.

A detailed review of the results revealed that none of the organs of any mink were CIEP positive when plasma antibody titers at 218 dpi were 16 or lower, and all organs of each mink, except one intestine sample, were CIEP positive when antibody titer was 64 and higher. The GENMOD analysis showed that the incidence of PCR‐positive spleen samples (0.878) was significantly greater than that in the liver and intestine samples, and that of the liver was significantly lower than that in all other organs (Table 9). There was no significant difference among the organs for the incidence of CIEP‐positive cases.

**Table 9:** Least square means ± SE of the proportion of PCR‐ and CIEP‐positive organs

PCR^d^	Mean ± SE^e^	CIEP	Mean ± SE^e^
Spleen	0.878 ± 0.051 a	Spleen^f^	0.707 ± 0.071
Liver	0.512 ± 0.078 b	Lymph nodes	0.676 ± 0.077
Kidneys	0.805 ± 0.062 ac	Intestine	0.683 ± 0.073
Lungs	0.781 ± 0.065 ac		
Intestine	0.585 ± 0.077 c		

Abbreviations: CIEP, counter‐immunoelectrophoresis; PCR, polymerase chain reaction.

^d^ All lymph nodes and bone marrow samples were PCR positive and could not be included in the model.

^e^ Means followed by different letters are different at *P* < .05.

^f^ CIEP results were the same in the spleen, liver, kidneys, and lungs of all mink.

### 3.5 AMDV DNA in saliva and rectal swabs

The incidence of PCR‐positive saliva samples was the highest at 20 dpi (0.89) and decreased to the lowest level (0.13) at 196 dpi (Table 10). The incidence of PCR‐positive rectal swabs increased from 20 dpi (0.40) to its highest level at 56 dpi (0.77) and then declined to 0.29 at 196 dpi. Estimates of kappa coefficients suggested that agreements between PCR results of the two sampling sites were moderate at 56 and 84 dpi, but weak on the other sampling occasions (Table 10). Estimates of sensitivity and specificity of PCR results of the saliva and rectal swabs, relative to plasma PCR, did not show clear patterns over time (Table 4). The sensitivity of saliva PCR was the highest at 20 dpi (0.85) and then decreased over time. On the contrary, the sensitivity of the rectal swab was the lowest at 20 dpi (0.52), gradually increased to 0.84 at 84 dpi, and then declined. Estimates of specificity of saliva relative to plasma PCR ranged between zero at 20 dpi and 1.0 at 35 dpi but estimates of specificity of rectal swabs were 1.0 at 20, 35, and 56 dpi, then declined.

**Table 10:** Incidence of PCR‐positive saliva and rectal swabs and the agreement between the results of the two sampling sites

	Saliva		Rectal		
Days postinoculation	No.	PCR positive	No.	PCR positive	Kappa coefficient
20	35	0.89	35	0.40	−0.04
35	35	0.57	35	0.69	0.28
56	34	0.79	35	0.77	0.40
84	32	0.53	33	0.70	0.42
140	30	0.20	31	0.45	−0.11
196	31	0.13	31	0.29	−0.03

Abbreviation: PCR, polymerase chain reaction.

Percentages of cases where both plasma and saliva were PCR positive and cases where plasma and rectal PCR were positive, were much lower at 140 and 196 dpi compared with the previous sampling occasions, whereas percentages of cases where both were PCR negative showed increasing trends by time. Kappa coefficients revealed that there were very weak agreements between plasma and saliva PCR in the same individuals on all sampling occasions, and those between plasma and rectal swab PCR results were intermediate at 84 and 140 dpi, but weak on other sampling occasions (Table 4).

### 3.6 Sprinklers

None of the mink exhibited high levels of white hair fibers (scores 3 or 4) and the prevalence of sprinklers with scores 1 and 2 at 196 dpi were 17.2% and 8.6% of 58 mink, respectively, and those with scores 1 and 2 at 218 dpi were both 21.9% of 41 mink. None of the mink inoculated with lower than 10^−4^ and 10^−5^ doses had sprinklers at 196 and 218 dpi, respectively (Table 11). The proportions of mink that showed sprinklers was almost three times greater in adults than in the juveniles at 196 (18.9% vs 6.9%, *χ* ^2^_(2)_ = 3.91, *P* = .048) and 218 dpi (31.7% vs 12.2%, *χ* ^2^_(2)_ = 3.46, *P* = .063). Spearman's rank correlation coefficient between sprinkler scores at 196 and 218 dpi was positive and large (*r* = .72, *P* < .01). Antibody titer on 218 dpi had weak relationships with sprinkler scores on 196 (*r* = .12) and 218 (*r* = .09) dpi.

**Table 11:** Percentage of mink showing sprinklers at 196 and 218 days postinoculation by inoculum dose

Days postinoculation	Score	Inoculum dose					
		10^0^	10^−1^	10^−2^	10^−3^	10^−4^	10^<−4^ ^a^
196	0	0.71	0.50	0.38	0.33	1.00	1.00
	1	0.14	0.50	0.25	0.50	0.00	0.00
	2	0.14	0.00	0.38	0.17	0.00	0.00
218	0	0.71	0.38	0.38	0.50	0.86	0.60
	1	0.14	0.50	0.25	0.17	0.14	0.00
	2	0.14	0.12	0.38	0.33	0.00	0.40
	No. of mink	7	8	8	6	7	22^b^

^a^ Animals inoculated with 10^−5^ and lower doses were combined.

^b^ There were 22 and 5 mink in this group at 196 and 218 dpi, respectively.

### 3.7 ID_50_

The estimates of ID_50_ of the 10% spleen homogenate based on CIEP and PCR results sharply decreased from 20 to 35 dpi but showed minor changes from 35 to 56 dpi (Table 12). The estimates based on CIEP were more than two times greater than those based on PCR results at 20, 35, and 56 dpi.

**Table 12:** Estimates of ID_50_ and their 95% confidence limit (in bracket) using plasma CIEP and PCR results at days 20, 35, and 56 postinoculation in mink inoculated with 0.5 mL of different dilutions of the10% spleen homogenate

Days postinoculation	CIEP	PCR
20	1.38 × 10^−2^ (2.0 × 10^−3^ – 1.8 × 10^−1^)	2.61 × 10^−3^ (4.3 × 10^−4^ – 1.9 × 10^−2^)
35	3.16 × 10^−4^ (3.8 × 10^−5^ – 2.6 × 10^−3^)	1.59 × 10^−4^ (1.0 × 10^−5^ – 2.0 × 10^−3^)
56	4.35 × 10^−4^ (4.9 × 10^−5^ – 4.0 × 10^−3^)	1.62 × 10^−4^ (1.3 × 10^−5^ – 1.7 × 10^−3^)

Abbreviations: CIEP, counter‐immunoelectrophoresis; PCR, polymerase chain reaction.

### 3.8 Control group

One control mink that was CIEP and PCR negative died after sampling at 84 dpi, for reasons not related to AMDV infection. One mink remained CIEP and PCR negative until 196 dpi, one became CIEP and PCR positive for the first time at 196 dpi, and one became PCR positive at 84 dpi and CIEP positive at 196 dpi.

## 4 DISCUSSION

Inoculating mink with 0.5 mL of the 10^0^ and 10^−1^ doses in the current study overwhelmed the host innate defense system and caused infection in all mink, whereas very low inoculum doses did not cause infection in most animals. It may be hypothesized that both of these extremes did not allow the manifestation of the genetic potential of the mink for expressing their responses to infection. The results agree with a previous report that the inoculation of mink with high doses of four viral strains varying in pathogenicity resulted in the same proportion of infection in pastel mink, whereas low inoculum doses caused variable responses among animals.^18^ Differences in viremia and antibody response among animals within each inoculum dose lower than 10^−1^ in the current study, which is in concordance with previous reports,^26^, ^27^ were the manifestation of genetic differences among individual mink and is the basis for the establishment of tolerant herds.

A notable finding in the current study was the significant decrease in antibody titer at 218 dpi by decreasing the inoculum dose. The observation that the effect of inoculum dose on antibody titer was primarily the consequence of the length of time elapsed between the establishment of infection, measured by viremia, and the time of assessing antibody titer (Table 5) is particularly important when antibody titer is used to identify tolerant mink. It may be concluded that antibody titer in chronically infected mink, where viral dose and the time of exposure to the virus are not known may not be an accurate indicator of tolerance to AMDV infection, and uniform inoculation of mink is needed. The results also imply that the identification of tolerant mink on infected farms based on a single measure of antibody titer may not be accurate because of differences among mink for the time of establishment of infection and that antibody titer in some animals may be elevated^28^ or reduced^7^, ^17^ over time.

The reduction in the rate of viral replication after 10 dpi^29^ causes irregular and short‐lived viremia in some mink, as was observed in the current and previous studies.^7^, ^10^, ^16^, ^28^ A large number (71.1%) of the inoculated mink in the current study had irregular or short‐lived viremia by 196 dpi, even though the virus was present in all lymphoid organs, which agrees with a previous study.^26^ This finding implies that PCR is not an accurate tool for detecting infection in chronically infected mink. Differences in the duration of viremia among mink inoculated with the same dose of a virus in the current and other studies^10^, ^28^ point to the importance of the host genetics on viral replication. It may be postulated that mink that have short‐lived or irregular viremia may be those which could tolerate the infection, whereas continuous viral replication and viremia may be associated with an increased risk for disease progression.^26^ If this hypothesis is correct, it may be concluded that the duration of viremia could be an accurate measure of tolerance, although this relationship needs to be fully investigated.

Persistence of antibody production in the current study, which was observed even after the termination of viremia, agrees with previous reports.^7^, ^16^, ^21^, ^26^, ^28^ A positive CIEP test suggests that the animal has been infected with the virus, but the virus may not be present in the bloodstream at the time of testing (PCR negative), a condition that was previously reported.^13^, ^30^ On the contrary, some infected mink are genetically prone to produce low antibody titers that are not detectable by CIEP,^8^ resulting in the presence of PCR‐positive plasma in seronegative mink.^13^, ^30^, ^31^ The observation that low inoculum doses significantly reduced the incidence of CIEP‐positive cases at all sampling occasions and that 43.1% of the mink that were inoculated with 10^−2^ and lower doses remained seronegative until 196 dpi suggest that animals on infected farms, which are naturally exposed to low doses of the virus, may be infected for sometimes before being detected by CIEP or may not be detected at all. This situation has important epidemiological ramifications for viral eradication programs and could be one of the reasons for the persistent infection of mink on farms that practice the test‐and‐cull strategy.^5^, ^6^ The observation that infection was detectable in blood by PCR earlier than antibodies by CIEP is in concordance with previous studies,^21^, ^23^, ^32^ and suggests that PCR is recommended for testing AMDV outbreak on virus‐free farms, but it is not accurate for testing infection in chronically infected mink.

The findings that the association between inoculum dose and the incidence of viremia was significant during the early sampling occasions but was no longer significant after 84 dpi (Table 2, Figure 2) was the consequence of a combination of short‐lived viremia and the establishment of infection via viral transmission from infected mink in the shed (secondary infection) in those mink that were not infected earlier with low doses of the virus. On the contrary, the observation that the associations between inoculum dose and the incidence of seropositive mink remained significant at all sampling occasions (Table 3, Figure 4) was the result of persistent antibody production, although increases in the incidence of secondary infections made this relationship weaker over time (Figure 4).

Differences between profiles of antibody production and viremia in each animal resulted in the sensitivity and specificity of CIEP to reveal different pictures of infection at various times postinoculation (Table 4). The production of detectable levels of antibodies later than the detection of infection by PCR resulted in false‐negative CIEP relative to PCR results, and thus low CIEP sensitivity at 20 dpi. Short‐lived viremia, persistent antibody production, and secondary infections resulted in fluctuations in sensitivity of CIEP over time. All CIEP‐positive mink at 20 dpi were also PCR positive, resulting in 100% specificity, whereas short‐lived viremia at later dates increased the incidence of positive CIEP and negative PCR (false positive), and a decreasing trend in the estimate of specificity. Comparable results were previously reported in mink tested on 10, 21, 36, and 56 dpi.^21^ These data suggest that the results of CIEP and PCR tests are influenced by the duration of time after infection and need to be interpreted with caution.

Animals that were tested at 218 dpi were all infected, as indicated by the presence of AMDV DNA in their lymphoid organs, which are the sites of viral replication.^33^ Detection of the virus in a smaller number of the liver, kidneys, lungs, and intestine samples than in the lymphoid organs agrees with previous reports.^21^, ^23^, ^26^, ^33^, ^34^ Comparable results were also reported in another study, except a much lower PCR‐positive bone marrow samples than the spleen and lymph nodes.^32^ The differences in the incidence of PCR‐positive cases among organs were possibly the result of reduced viral replication, which resulted in decreases in viral load in organs^28^, ^29^ along with the presence of variable amounts of substances in different organs that reduce or inhibit PCR amplification.^35^, ^36^ The inoculum dose had a significant effect only on the incidence of PCR‐positive liver samples (Table 8), which was possibly because of the low incidence of PCR‐positive liver samples (Table 9), whereas large numbers of other organs, regardless of the inoculum doses, were PCR positive.

The presence of antibody titers in organs was measured to test whether CIEP can be used to detect infection in mink cadavers. The findings that detectable levels of antibodies were present at 218 dpi in all six organs of all mink inoculated with 10^0^ and 10^−1^ doses, and the significant effect of inoculum dose on the incidence of CIEP‐positive cases in all organs (Table 8) point to the strong and positive associations between plasma antibody titer and the level of antibodies in organs. This relationship was confirmed by the observation that none of the organs of any mink was CIEP positive when plasma antibody titers were 16 or lower, and all organs of all mink were CIEP positive when antibody titer was 64 and higher. The results are in concordance with previous reports where antibody was not detected in any of the seven organs of mink on 10 dpi when antibody titer was low,^23^ but was detected at 56 dpi in all five organs of almost all mink inoculated intranasally after sedation or intraperitoneally.^21^ It can be concluded that the CIEP test in organ homogenates is generally unreliable for detecting AMDV infection in mink cadavers.

Mink that were exposed to low doses of the virus mostly seroconverted by 84 dpi (corresponding to October) or later, as a result of secondary infection. Increases in the incidence of seropositive mink from summer to late fall were previously reported,^11^, ^12^ which was possibly attributed to a combination of a gradual increase in antibody titer,^28^ or stress caused by hormonal changes during molting,^37^ handling mink for pelt evaluation or cold weather. It can also be the result of increased secondary infection because of a more efficient viral transmission during cold weather. The significantly greater proportion of PCR and CIEP positive adult than juvenile mink at 20 dpi, which disappeared at later dates (Tables 2 and 3), could be the result of a more robust immune system of juveniles that delayed the establishment of infection by a yet unexplored reason. Maternal immunity did not play a role in this finding because juveniles were obtained from AMDV‐free farms.

Toenail clipping is stressful for mink and increases the risk of viral transmission among animals^16^ because using a new clipper for each mink is not practical and is seldom implemented on farms. Any sampling method that circumvents toenail clipping is attractive to farmers, particularly when continuous monitoring of infection status of the mink is required. Taking rectal and fecal samples would also be less stressful on conscious mink than saliva sampling, which requires the difficult task of opening the mouth. Furthermore, the mouths of sedated mink become dry, making it difficult to collect enough saliva.^23^ The estimates of the sensitivity of saliva and rectal swabs showed inconsistent and somewhat unreliable results when compared with plasma PCR (Table 4), which agrees with previous studies for saliva and rectal swabs^21^, ^23^ and for oronasal and fecal samples.^32^ The steady decrease in the incidence of PCR‐positive saliva samples over time (Table 10) along with irregular viremia were manifested in a downward and fluctuating trend in the false‐negative saliva PCR results, and consequently a similar trend in the estimates of sensitivity (Table 4). The increase in the incidence of PCR‐positive rectal swabs from 20 to 56 dpi, and its subsequent decline over time (Table 10) reflected in the instabilities in the estimates of sensitivity of the rectal swabs. The observation that except for saliva at 20 dpi, estimates of specificity were generally high and showed slight declines over time agree with previous reports.^21^, ^23^ Changes in the incidence of PCR‐positive saliva and rectal swabs were the manifestation of decreases in viral replication and reduced viral load in the circulation over time,^29^ along with the presence of inhibitors, such as bile salts and polysaccharides in feces and polysaccharides in saliva, which were not removed by the DNA extraction process, and could have reduced PCR amplification success.^35^, ^36^ The use of saliva and rectal swabs in viral eradication programs are thus not recommended because of the low estimates of sensitivity during the late periods of infection. The false‐negative tests in these sampling sites would result in the failure of eradication programs. The estimates of specificity of the saliva and rectal swabs were larger than the estimates of sensitivity, as a result of low number of false‐positive cases. In practice, this situation would result in the culling of some noninfected mink with no impact on the success of viral eradication programs.

Infection with AMDV causes depigmentation of hair fibers, which drastically reduces the market value of the pelt. In one study, all 17 naturally infected black mink with sprinklers had histopathological lesions of AD and harbored AMDV DNA when tested in late October.^13^ In another study, 2 of the 12 wild‐type mink inoculated with AMDV showed sprinklers at 24 weeks pi, but none of the other 23 mink tested at 8‐ and 16‐week pi showed sprinklers.^19^ The observation that the incidence of sprinklers was not related to antibody titer in the current study but was detected only in the mink inoculated with 10^−4^ and higher doses (Table 11), could imply that the duration of infection had the greatest effect on the incidence of sprinklers. The greater proportion of adults with sprinklers than juveniles in the current study, which was parallel with the differences between these two age groups for the prevalence of infection at 20 dpi, is further evidence that the duration of infection was an important factor in the development of sprinklers. Mink genotype also plays a role in the development of sprinklers because in the current and the other study^19^ only a fraction of animals, which were inoculated at the same time, showed sprinklers. It was hypothesized^38^ that hair depigmentation is possibly caused by a disruption of the melanin production in hair follicles due to the death or malfunctioning of melanocytes or the disruption of the melanin pathway.^39^ AMDV infection causes elevated levels of the Th1 and Th2 cytokines,^40^, ^41^ which may result in the death of melanocytes, similar to the depigmentation in patches of skin in humans with vitiligo disorder.^39^

Very high doses of the inoculum, often several hundred times greater than ID_50_ are used when the guaranteed establishment of infection is required. Exposure to very high doses of the virus, however, does not naturally occur on farms and conceals the expression of genetic differences among individuals in the course of infection. Identification of tolerant mink requires exposure at a level that permits the manifestation of the animals' genetic potential, that is, less than 0.5 mL of the 10^−1^ dilution of the 10% spleen homogenate used in the current study. Differences between the estimates of ID_50_ based on CIEP and PCR were much smaller at 20 dpi compared with those at 35 and 56 dpi, which were somewhat comparable. The results suggested that it is difficult to have a single estimate of ID_50_ for all sampling occasions and for both methods of measuring infection. Such changes are logical because of the differences between profiles of antibody production and viral replication over time. Testing of mink for infection by CIEP at 20 dpi may not be accurate because of the delay in the production of detectable levels of antibodies. Because a high proportion of animals were PCR and CIEP positive at 35 dpi in the current study and 36 dpi was the earliest time when infection was detected by both PCR and CIEP in plasma of mink inoculated intranasally after sedation,^21^ and because the estimates of ID_50_ did not show much change between 35 and 56 dpi based on the CIEP or PCR tests, it was concluded that 35 or 56 dpi are logical times to test for the presence of infection. Both short‐lived viremia and secondary infections add inaccuracies in estimating ID_50_ of an inoculum after 56 dpi.

The finding that two of the three control mink became infected after 84 dpi is evidence that natural transmission of AMDV causes infection in most mink housed in a contaminated environment. In a previous experiment, AMDV DNA was detected in the lymph nodes of three control mink at 56 dpi, whereas viral DNA was detected in plasma of two and none was seropositive by this time.^21^ In another experiment, four of the six control mink became infected 6 to 7 weeks after other mink in the same room were inoculated.^32^ The slow rate of horizontal AMDV transmission in an infected environment^11^, ^16^ and the variable response of mink to natural infection, in combination with the delay in detecting antibodies in plasma by CIEP^21^, ^23^, ^27^, ^32^ are reasons for the failure of the test‐and‐removal strategy. The observation that only a fraction of mink becomes naturally infected, and the time of infection varies widely among mink, causes inaccuracies in the assessment of tolerance when antibody‐based tools are employed on naturally infected mink farms. These observations are another reason that accurate identification of tolerant mink to AMDV infection requires uniform exposure of all mink in herds with the virus.

## 5 CONCLUSIONS

Persistent antibody production and short‐lived and irregular viremia confirmed that CIEP is more accurate than PCR for detecting infection on chronically infected mink farms and is the preferred tool for virus eradication programs. Contrary to seroconversion, which was significantly associated with the inoculum dose at all sampling occasions, the association between inoculum dose and the incidence of viremia was not significant after 84 dpi. It was hypothesized that mink with short‐lived or irregular viremia may be those which could tolerate the infection, whereas continuous viral replication and viremia may be associated with an increased risk for disease progression, although this hypothesis needs further investigation. Antibody titer at 218 dpi significantly decreased with decreasing inoculum dose, which was primarily the consequence of the time elapsed between the establishment of infection, measured by viremia, and the assessment of antibody titer. This relationship is particularly important when antibody titer is used to identify tolerant mink on naturally infected farms. This observation implies that because infection occurs at various times on a naturally infected farm, the identification of tolerant mink based on antibody titer is subject to inaccuracies and exposure of the entire herd to an infective dose of the virus via a natural route is needed. Juvenile mink were able to delay the establishment of infection, thus a significantly greater proportion of adults than juveniles became PCR and CIEP positive at 20 dpi, but the differences disappeared on subsequent sampling occasions. Age of mink did not affect antibody titer at 218 dpi either, suggesting that the same method of evaluation of tolerance can be used for juveniles and adults. PCR tests on the saliva and rectal swabs were inconsistent and somewhat unreliable when compared with plasma PCR results.

## CONFLICT OF INTERESTS

The authors declare that there are no conflict of interests.

## AUTHOR CONTRIBUTIONS

AHF and IH participated in the research design. IH performed animal inoculation and sampling. AHF performed data analysis and prepared a draft of the manuscript. IH critically reviewed the manuscript.

## Data Availability

The data that support the findings of this study are available from the corresponding author upon reasonable request.
